# A qualitative exploration of community mobility experiences of wheelchair users

**DOI:** 10.4102/ajod.v13i0.1253

**Published:** 2024-02-16

**Authors:** Jerome P. Fredericks, Surona Visagie, Lana van Niekerk

**Affiliations:** 1 Division of Occupational Therapy, Department of Health and Rehabilitation Sciences, Faculty of Medicine and Health Sciences, Stellenbosch University, Cape Town, South Africa; 2 Centre for Disability and Rehabilitation studies, Faculty of Medicine and Health Sciences, Stellenbosch University, Cape Town, South Africa

**Keywords:** wheelchair users, minibus taxis, community mobility, barriers, experiences, taxi drivers, minibus taxi drivers, natural manmade and environmental barriers

## Abstract

**Background:**

Freedom of movement, which is dependent on community mobility, is a key contributor to good quality of life and important in the establishment of a person’s community identity.

**Objective:**

To describe the community mobility experiences of wheelchair users who lived in a socio-economically challenged setting.

**Method:**

The study setting was Paarl, a peri-urban area of the Western Cape province of South Africa. This article reports findings from phase 1 (a reflection on past community mobility and minibus taxi use experiences) of cycle 1 of a co-operative inquiry. Nine adult wheelchair users, eight caregivers, six minibus taxi drivers, and four community stakeholders participated. Data were collected during a focus group discussion and analysed using inductive thematic analysis.

**Results:**

Four themes, ‘Knowledge, attitudes, and actions’, ‘Natural, manmade and mechanical environmental barriers’, ‘Health and safety concerns’ and ‘Poor community participation and quality of life’ were identified. The themes showed how difficult an everyday activity like moving around in the community were for wheelchair users, and how that limited their community involvement.

**Conclusions:**

Wheelchair users living in a low-income peri-urban area struggled to participate in community activities meaningful to them because various barriers hampered community wheelchair mobility and minibus taxi use.

**Contribution:**

The findings regarding community mobility struggles and specifically minibus taxi access guided specific recommendations and the further phases and cycles of the co-operative inquiry. The purpose of the co-operative inquiry was to allow co-researchers to find their voice and develop solutions to minibus taxi access for wheelchair users.

## Introduction

All people, including wheelchair users, have the right to freedom of movement, that is, moving freely in their own country and between countries as well as changing living and/or employment spaces (Rosenfeld 2020). Freedom of movement is a key contributor to good quality of life and important in the establishment of a person’s identity within their communities (Pyer & Tucker 2017). Community mobility is essential to realising the right to freedom of movement. Driving and community mobility are defined within the Occupational Therapy Practice Framework: Domain and Process, in the following way:

[*P*]lanning and moving around in the community and using public or private transportation, such as driving, walking, bicycling, or accessing and riding in buses, taxi cabs, or other transportation systems. (American Occupational Therapy Association [AOTA] 2014:S19)

Community mobility underpins many aspects of life as it is a prerequisite for community participation (Poole et al. 2018). Humans cannot connect with others, places of occupation, and contribute to community culture and functioning without community mobility (Bezyak, Sabella & Gattis 2017). Community mobility supports natural engagements in community-based contexts in different life domains, including domestic, interpersonal, education, employment, civic, religious, sport, leisure, and social life (World Health Organization [WHO] 2001). Spending time in the community, participating in social, leisure, recreational and civic activities, all lead to a greater sense of fulfilment (Seekins et al. 2007), and have a positive impact on quality of life.

Community mobility has a positive effect on physical and mental health (Brusilovskiy et al. 2020; Crabtree 2017; Zhu & Fan 2018) as it provides a sense of freedom, independence and self-worth (Bourret et al. 2002; Umstattd Meyer et al. 2014). Lower levels of community mobility lead to decreased social contact, lower levels of physical activity and social exclusion (Church, Frost & Sullivan 2000; Fristedt et al. 2014; Umstattd Meyer et al. 2014). A decrease in time spent outside the home has been connected to an increase in depressed mood and a greater prevalence of depressive symptoms (Petersen et al. 2015; Tsunoda et al. 2015). Physical deterioration, increased utilisation of healthcare services and premature death have also been linked to lower levels of community mobility (Gill et al. 2016).

Community participation by wheelchair users has been shown to be lower than that of ambulant persons (Carpenter et al. 2007; Harris 2007). Decreased community participation has a negative effect on the physical and mental health of wheelchair users (Strohle 2009; Zabriskie, Lundberg & Groff 2005). Without barrier-free access to their communities, wheelchair users might never be able to come out from their homes to transform resources into personal, social and professional achievements (Raja, Boyce & Boyce 2008). They will remain invisible, unable to contribute to or benefit from services and commercial activities that are available to citizens. Safe, affordable, physically accessible and acceptable community mobility options will enhance wheelchair users’ participation in society (Bezyak et al. 2017), which can lead to more independent and economically productive lives.

However, Park et al. (2023) through a systematic review on disability and travel behaviour, have shown that persons with disabilities (including mobility impairments) make two to four fewer trips per week than their nondisabled peers. They also on average travel significantly shorter distances (Park et al. 2023). Inaccessibility of community settings, challenges to get to public transport stops, decreased vehicle access, attitudinal barriers from drivers and fellow commuters, fear of injury, unsafe spaces, anxiety about the trip, the need for careful planning as well as physical weakness and diminished stamina all interacted to limit independence, spontaneity, and freedom to access the community (Park et al. 2023).

An earlier systematic review by Unsworth et al. (2019) focussed specifically on public transport access for persons with mobility impairments, showed similar findings. Unsworth et al. (2019) found that travelling in a wheelchair was hampered by uneven pavements, no dropped kerbs, steps and pedestrian traffic light controls being too high. The review went on to focus on access to kneeling buses, a facility unavailable in the current study setting. The limited space to manoeuvre inside buses, trams and trains was also described. However, none of the 26 studies reviewed by Unsworth et al. (2019) or the 115 reviewed by Park et al. (2023) were done in Africa.

Duri and Luke (2022a) explored transport barriers experienced by Africans with disabilities through a desktop literature review. They identified inaccessible transport infrastructure, poor maintenance of existing infrastructure as well as a lack and poor implementation of policy and legislation to be hampering transport access for persons with disabilities. They did not provide information on trip frequency or preferred means of transport. In a study conducted by Visagie et al. (2023), in a town neighbouring the current study setting, wheelchair users indicated that they use their wheelchairs to access their communities in addition to minibus taxis and privately owned vehicles. Some preferred their wheelchairs to other modes of community mobility, similar to findings in a Nigerian study by Bombom and Abdullahi (2016) which specifically mentions road safety and that the disregard motorists showed them were hazardous. Negative attitudes of drivers and fellow commuters were identified by all three reviews referred to above (Duri & Luke 2022a; Park et al. 2023; Unsworth et al. 2019). Taxi drivers, wheelchair users, and their assistants might also lack the knowledge and skills related to getting into or out of taxis, which might lead to frustration, embarrassment and injuries of wheelchair users during transfers (Duri & Luke 2022a).

In 1996, the White Paper on National Transport Policy had already stipulated that transport for persons with disabilities must be addressed in South Africa (Department of Transport 2000). Similarly, the *National Land Transport Transition Act 22 of 2000* advocates that public transport systems should cater to the accessibility requirements of persons with disabilities (Department of Transport 2000). More recently, the White Paper on the Rights of Persons with Disabilities stated that transport should be universally accessible in South Africa to create a free and just society inclusive of persons with disabilities (Department of Social Development 2016). However, despite policies and legislation advocating for inclusive transport, there is still a general neglect of the challenges confronting wheelchair users regarding public transport access in South Africa (Cawood & Visagie 2015; Lister & Dhunpath 2016; Vergunst et al. 2015). Inaccessible public and private transportation across the travel value chain remains a major barrier to the right to equality for wheelchair users in South Africa (Department of Social Development 2016).

Factors that negatively impact access to minibus taxis for wheelchair users relate to physical access, safety, cost and acceptability (Cawood & Visagie 2015; Lister & Dhunpath 2016; Vergunst et al. 2015; Visagie et al. 2023). The design of minibus taxis, such as the height difference between the wheelchair and the seat, makes it difficult for wheelchair users to board and disembark (Gudwana 2019; Pretorius & Steadman 2018; Vanderschuren, Baufeldt & Phayane 2019; Visagie et al. 2023). Inside the taxi, there is little space to manoeuvre (Vanderschuren et al. 2021) or store a wheelchair (Gudwana 2019). Lack of seat belts, reckless driving, overloading and an inability of the wheelchair user to maintain balance, all lead to a sense of feeling unsafe when using minibus taxis (Gudwana 2019; Kett, Cole & Turner 2020; Lister & Dhunpath 2016). It has been widely reported that taxi drivers’ attitudes, and their tendency not to stop for wheelchair users, are financially driven, because the taxi operators and owners’ attitudes are that ‘time is money’. Taxi operators’ wages and the profits made by the taxi owner depend on the number of passengers transported per day. Thus, overloading and exceeding the speed limit are common practices that are followed to increase income and profit (Lister & Dhunpath 2016). Furthermore, elderly people, children and persons with disabilities might be disregarded as they might take longer to embark and ‘waste time’ thereby decreasing profit (Lister & Dhunpath 2016). When using minibus taxis, wheelchair users are required to pay an additional fee for their wheelchairs which take up space that could be used for paying customers (Cawood & Visagie 2015; Grut et al. 2012; Mudzi, Stewart & Musenge 2013; Venter et al. 2002; Vergunst et al. 2015). Taxi drivers might be unwilling to assist wheelchair users and have an impatient attitude (Bombom & Abdullahi 2016; Cawood & Visagie 2015; Gudwana 2019; Lister & Dhunpath 2016; Visagie et al. 2023). Similarly, fellow travellers are in a hurry and disinclined to assist a wheelchair user or to wait while a wheelchair user gets on or off a taxi (Bombom & Abdullahi 2016; Duri & Luke 2022a; Visagie et al. 2023). Sometimes, wheelchair users have to wait longer than ambulant passengers in inconvenient, unsafe circumstances at taxi ranks (Venter Mahendra & Hidalgo 2019).

Both wheelchairs and vehicles, such as minibus taxis in the case of the current study, can provide community mobility. In both instances, an appropriate wheelchair plays a key role in ensuring mobility. A wheelchair unsuitable for the terrain or unsuitable to be loaded in a taxi will hamper community mobility. An appropriate wheelchair as defined by WHO (2008) and re-iterated in 2023 (WHO 2023) is one that:

[*M*]eets the user’s needs and environmental conditions, provides proper fit and postural support, is safe and durable, is available in the country; and can be obtained and maintained and services sustained in the country at an affordable cost. (WHO 2008:11)

The challenge is that an appropriate wheelchair for self-propulsion over peri-urban and rural terrain in South Africa is not always suited to easy loading and unloading in vehicles (Visagie et al. 2015). A lack of knowledge on the side of service providers further hampers prescription of appropriate wheelchairs (Visagie, Scheffler & Schneider 2013). In addition, wheelchair provision in South Africa is often dictated by available funding (Visagie et al. 2013; Visagie, Duffield & Unger 2015) rather than the four most appropriate wheelchairs. South Africans in need of wheelchairs are often provided with a basic four-wheel folding frame wheelchair with little consideration of appropriateness beyond affordable costs (Visagie et al. 2015). This model wheelchair should only be considered for temporary and short-term use as it is unsuitable for long term and/or active use in the community.

This article aims to build on previous research and describe the community mobility experiences of a specific group of wheelchair users who live in a lower socio-economic setting. It draws from a larger study with the aim to develop strategies to enhance wheelchair users’ access to minibus taxis. The study followed a co-operative inquiry approach with a focus on social justice. As such it was underscored by the social approach to disability, which promotes inclusion and social change through the transformation of society and advocates for the removal of social barriers (Shakespeare 2018). Specifically, the current co-operative inquiry attempted to promote inclusion of the wheelchair users by facilitating social change and overcoming social barriers through developing strategies to enhance accessibility of minibus taxi services for wheelchair users.

## Research methods and design

### Study design

**FIGURE 1:**
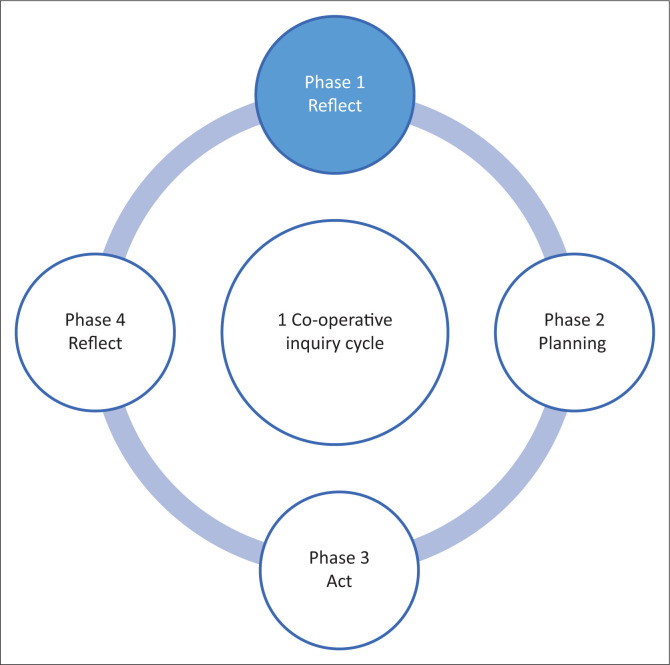
Diagrammatic presentation of phase 1 co-operative inquiry cycle.

The article reports on the first phase of the first cycle of a cooperative inquiry that was undertaken with wheelchair users and minibus taxi drivers as co-researchers. Within a cooperative inquiry design, disempowered persons are given a voice and the power imbalance traditionally found between academic researchers and study participations from disadvantaged groups is reduced (Wooltorton et al. 2020). Co-operative inquiry typically entails four distinct phases, which together form one research cycle. Each cycle starts with a reflective phase, followed by an active phase, a reflective review phase, and the fourth phase which is to plan the next action phase (Figure 1) (Wooltorton et al. 2020). In this study, co-researchers developed strategies on accessible minibus taxi services for wheelchair users over four cycles, at which time they were satisfied with the outcomes.

The co-operative inquiry started with an introduction session where the study purpose and activities as well as the role of co-researchers were explained to potential participants. After this explanation, those interested to participate signed an informed consent form. Those not interested left the meeting. Co-researchers then introduced themselves to each other, discussed the co-operative inquiry purposes and processes, developed a group contract, and assigned roles such as scribes and timekeepers among themselves.

In phase one of the cycle, one co-researchers shared experiences on using minibus taxis and providing transport to wheelchair users respectively. The current article reports on these findings (Figure 1). A previous paper described the actual methods used, including the informed consent process, how co-researchers were introduced to each other and the study, role division, and the development of a contract among co-researchers as well as how co-operative inquiry can serve as a strategy to empower marginalised groups. The strategies that were developed will be described in a future paper.

### Study setting

The current study was done in Paarl East, a low income, peri-urban area of the Western Cape Province of South Africa. The area is known for rising unemployment, economic downturn, job losses, crime, high rates of teenage pregnancies and high school dropouts. Drug-related crimes that affect all aspects of society, for example, family structures, health, the work environment, and the economy, are common in the area (Drakenstein Municipality 2017). Paarl East community members mainly walk or use minibus taxis to access their community.

South African minibus taxi services are privately owned and operated, with little fixed infrastructure, such as routes and time schedules. Services provided by these taxis are not as regulated as government-funded public transport, and taxi owners are not obligated by law to follow universal access principles (Lister & Dhunpath 2016; National Department of Transport 2000). They might choose not to provide services to persons with disabilities (Lister & Dunpath 2016).

As far as the authors know no strategies to enhance accessibility of minibus taxis have been tried in the area. Co-researchers also pointed out that the inquiry was a first for the Paarl area because in the past nothing had been done to address this issue.

**FIGURE 2:**
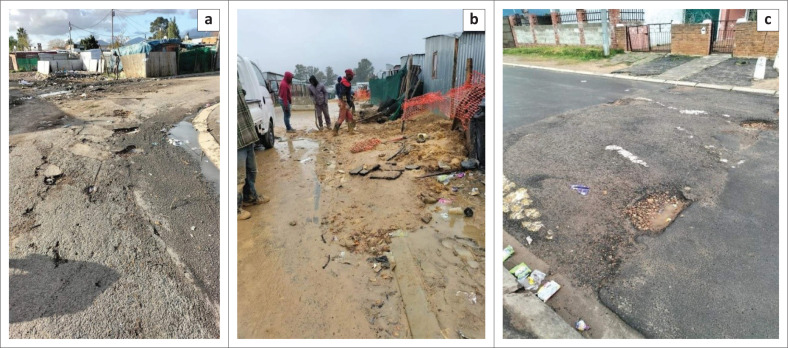
Photos (a, b, c) showing the streets and pavements in the study setting in dry and wet weather.

Paarl is situated among mountain ranges and thus the terrain is hilly with steep inclines. The town has a Mediterranean climate with wet, cold winters and dry, hot summers. Not all roads and pavements in the town are tarred. Some of the tarred roads have potholes. Untarred roads in the informal settlements are muddy with pools of water on rainy days. When dry, the ruts caused by vehicles driving on the wet dirt roads and potholes caused by puddles of water, remain. Figure 2 provides visual information on the condition of roads and pavements in the study setting.

### Sampling and recruitment

Co-operative inquiry requires a substantial time commitment and high level of involvement from participants. Therefore, adult wheelchair users, their caregivers and minibus taxi-drivers, living in the study setting, who were willing and able to make this commitment were sought. Wheelchair users had to use or wanted to use minibus taxi services. Wheelchair users included both those who were independent in the community and those who required assistance. Minibus taxi drivers had to have a public transport licence and taxi permit to transport people.

Twenty-three wheelchair users were contacted, through disabled people’s organisations and by approaching wheelchair users in the community. Nine consented to participate in the study. Eight caregivers (one wheelchair user did not have a caregiver) also participated.

The chairperson of the Paarl Taxi Association Group referred the lead researcher to 19 minibus taxi drivers of whom 7 provided consent to participate in the study. The other participants were the 4 stakeholders involved in disability matters in the area and consisted of a professional nurse, a disability activist, a wheelchair repairman and an interested community member, respectively. The co-operative inquiry group was completed by the lead researcher who is also the first author of this article and a research assistant.

### Data collection

The cooperative inquiry was done between June and December 2021. The reflection session reported on in this paper was held on 15 June 2021. Participants were asked to reflect on the best and worst taxi services they had experienced as wheelchair users or provided to wheelchair users as taxi drivers. Wheelchair users’ experiences of moving in the community using their wheelchairs were also explored.

The session lasted 180 min and was conducted in Afrikaans, the participants’ home language, and the preference of all group members. This and the other sessions were guided by the lead researcher, but all group members were treated as equals, and all had the opportunity to speak their mind. The group contract which addressed issues like cell phone use, timeliness, confidentiality and respect assisted in developing a sense of equality. So did sharing a free cooked meal after sessions. The sessions were digitally audio-recorded, and four sets of field notes were kept by volunteers from among the group.

Group members also communicated on the WhatsApp platform. These messages were included as data with their permission. One of the group members was illiterate. His caregiver read the information to him. Accommodations were further made by communicating on the WhatsApp platform with voice notes.

### Data management and analysis

**FIGURE 3:**
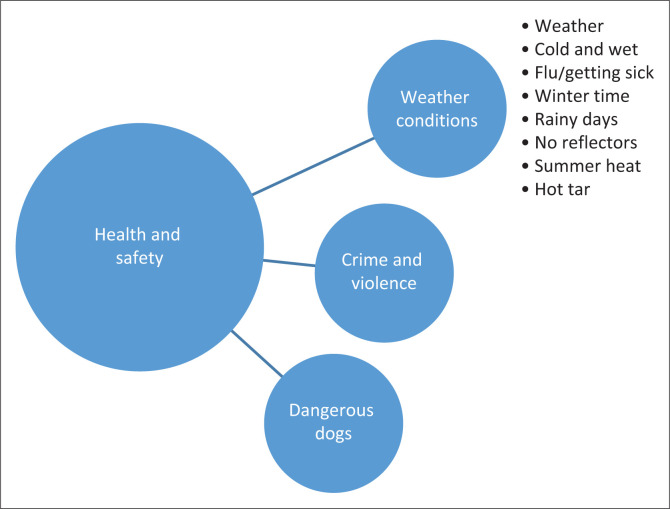
Thematic presentation of move from codes to a theme during data analysis.

Data were transcribed verbatim and analysed using the six steps of Braun and Clarke’s (2012) inductive thematic analysis approach. The first author coded the data manually, by reading the transcribed data line by line and assigning codes. Codes described what was seen in the data, and the data were organised into meaningful groups such as stairs in house, distances, weather, dogs, violence and disrespect. The codes and all supporting quotes were organised into provisional themes. Figure 3 shows the process followed from one group of codes to a category to a theme. This provisional analysis was shared with the second author who further developed the themes, and the two authors reached consensus on the final themes.

### Trustworthiness

Sharing in a group, where co-researchers could affirm each other, enhanced the credibility of findings. A further strength of the study was the inclusion of a research assistant who was known to the community and skilled in qualitative research. His skilful facilitation enhanced participation of group members and the quality and richness of data as also described by Flenady et al. (2022). The research assistant previously worked as a physiotherapist in the community. At the time of the co-operative inquiry, he was the chairperson of the Foundation for the Communities of Excellence youth programme to prepare high school learners for tertiary education. To support transferability, a detailed description of the research setting, methods and participants’ demographics was provided.

### Ethical considerations

The Health Research Ethics Committee of Stellenbosch University (S21/01/009) provided ethical approval. The amount of time and level of commitment required from participants were made clear in the informed consent form and during an introduction meeting. Written consent was provided by all participants. Participants were compensated for their time through cash payments. Data were stored in a password-protected Stellenbosch University’s SUNScholar Research Repository, where it will be kept for 5 years.

## Findings

Demographic details: The nine wheelchair user participants were mostly males aged between 32 and 67 years (Table 1). All had acquired injuries at different times in their lifetimes, and used manual, four-wheel folding frame wheelchair without adjustability features. Five out of the nine wheelchair users could propel themselves in the community. All nine wheelchair users were dependent on assistance when making use of minibus taxi services. Some wheelchair users propelled with two hands, other used one hand and one foot to propel. The foot propellers were able to use their wheelchairs independently in the community.

**TABLE 1:** Demographic details of wheelchair users.

Participant number	Gender	Age	Diagnosis	Self-propelled orattended propelled	Dependent on assistanceto use minibus taxi	Occupation	Income: Source/ amount per month	Marital status
WCU1	Male	50	Spinal cord injury	Self-propelled	Dependent	Unemployed	Social grant R2080 US$117.64	Married
WCU 2	Female	49	Cerebral vascular accident	Self-propelled withhand and foot	Dependent	Unemployed	Social grant R2080 US$117.64	Married
WCU 3	Male	52	Cerebral vascular accident	Self-propelled withhand and foot	Dependent	Unemployed	Social grant R2080 US$117.64	Married
WCU 4	Male	57	Cerebral vascular accident	Attended propelled	Dependent	Unemployed	Social grant R2080 US$117.64	Single
WCU 5	Male	55	Spinal cord injury	Self-propelled	Dependent	Unemployed	Social grant R2080 US$117.64	Married
WCU 6	Male	32	Spinal cord injury	Attended propelled	Dependent	Unemployed	Social grant R2080 US$117.64	Married
WCU 7	Female	54	Amputation of the lowerlimb.	Self-propelled	Dependent	Unemployed	Social grant R2080 US$117.64	Single
WCU 8	Male	67	Spinal cord injury	Attended propelled	Dependent	Unemployed	Social grant R2080 US$117.64	Married
WCU 9	Male	37	Spinal cord injury	Attended propelled	Dependent	Employed	SAP R15 533.97 US$803.91	Married

Table 2 shows that caregivers’ ages varied between 28 and 67 years. Six of them were women. Taxi drivers were in their 30s or 40s.

**TABLE 2:** Demographic details of other co-researchers.

Participant number	Gender	Age in years	Profession	Income source	Amount per month	Marital status
**Caregivers (C)**						
C1	Female	63	Retired	Social grant	R2080 $109.23 (USD)	Divorced
C2	Male	51	Employed	Tradesman	R1 090 055 US$578.82	Married
C3	Female	45	Unemployed	No income	-	Single
C4	Male	28	Unemployed	No income	-	Single
C5	Female	48	Unemployed	No income	-	Married
C6	Female	67	Retired	Social grant	R2080 $109.23 (USD)	Married
C7	Female	55	Unemployed	No income	-	Married
C8	Female	41	Employed	Casual worker	Not disclosed	Married
**Minibus taxi drivers (TD)**						
TD1	Male	44	Employed	Taxi driver	R846 703 US$449.6	Married
TD2	Male	32	Employed	Taxi driver	R846 703 US$449.6	Single
TD3	Male	48	Employed	Taxi driver	R846 703 US$449.6	Married
TD4	Male	45	Employed	Taxi driver	R846 703 US$449.6	Married
TD5	Male	48	Employed	Taxi owner/driver	Not disclosed	Married
TD6	Male	38	Employed	Taxi driver	R846 703 US$449.6	Single
TD7	Male	37	Employed	Taxi driver	R846 703 US$449.6	Single
**Other (S = Stakeholder)**						
S 1	Male	66	Retired	Social grant	R2080 $109.23 (USD)	Single
S 2	Female	62	Retired	Social grant	R2080 $109.23 (USD)	Divorced
S 3	Male	43	Employed	N/A	Not disclosed	Married
S 4	Male	34	Employed	N/A	Not disclosed	Married
Lead researcher	Male	45	Employed	-	Not disclosed	Married
Research Assistant	Male	50	Employed	-	Not disclosed	Married

### Emergent themes

Four themes emerged for the data as shown in Table 3. Theme 1, ‘Knowledge, attitudes, and actions’ shows that minibus taxi drivers lack the knowledge and skills to transfer wheelchair users into and out of a minibus taxi. It also presents a lack of mutual respect between drivers, wheelchair users and fellow commuters. Theme 1 ends with describing how some minibus taxi drivers did provide transport to wheelchair users while others refused. Theme 2, ‘Natural, manmade, and mechanical environmental barriers’, shows that aspects related to the physical environment such as the hilly nature of the town, dirt roads, and the design of minibus taxis resulted in barriers. Theme 3, ‘Health and safety concerns’, illustrates how a seemingly everyday activity, like moving around in the community could be challenging, with a negative impact on health and wellbeing. Theme 4, ‘Poor community participation and quality of life’, shows how community mobility challenges prevented wheelchair users and some caregivers from participating in life roles in the community.

**TABLE 3:** Themes and categories that emerged from the data.

Theme 1 Knowledge, attitudes and actions	Theme 2: Natural, manmadeand mechanical environmental barriers	Theme 3: Health and safety concerns	Theme 4: Poor community participation and quality of life
Knowledge. Attitude. Action.	Natural environmental barriers. Manmade environmental barriers. Mechanical environmental barriers.	Weather conditions. Crime and violence. Dangerous. Reckless driving No seat belts.	Unable to participate in valued activities. Negative effects on quality of caregiver lifestyle.

### Theme 1: Knowledge, attitudes and actions

Insufficient knowledge and awareness on the side of minibus taxi drivers about the needs of wheelchair users and more specifically how to physically assist them could have possibly led to attitudinal barriers.

#### Knowledge

Minibus taxi drivers shared that they did not have adequate knowledge about how to transfer wheelchair users and how to manage the wheelchair itself. This caused fear among some of them and made them chose to ignore wheelchair users rather than pick them up.

‘My problem is that I am not trained to transfer wheelchair users and what will happen if I am responsible for hurting a wheelchair user? They will sue me, and I don’t want that type of complication.’ (TD3 48, male)

‘The reasons why I don’t want to transport wheelchair users is that I don’t know how to transfer them. I also don’t know how to fold up a wheelchair. And some of their wheelchairs lack maintenance and I don’t want to struggle with their wheelchair which will take extra time if one has difficulty to get the wheelchair into the minibus taxis. As you know in our business time is money.’ (TD2 32, male)

Wheelchair user 2 agreed that minibus taxi drivers did not have an adequate understanding of their abilities and what support they need.

‘Minibus taxi drivers have no understanding when it comes to the transport of wheelchair users. It seems they lack insight into the medical condition of wheelchair users and will make statements like you can help yourself to get into the minibus taxi. It is disgraceful when you are being handled in such a negative way.’ (WCU2 49, female)

Wheelchair users experienced negative behaviour fuelled by lack of knowledge from fellow commuters. Wheelchair user 9 explained:

‘Fellow-commuters don’t have insight when it comes to wheelchair users. I am on medication for high blood pressure, consequently I need to use the bathroom frequently. On this day I wet my pants inside the minibus taxi, which was very embarrassing to me. The comments made by the co-commuters about me wetting my pants made it even more painful. I just wish they had better insight into my condition.’ (WCU9 37, male)

The lack of disability-related knowledge might have negatively influenced the relationship between the wheelchair users and minibus taxi drivers and facilitated the development of negative attitudes.

#### Attitudes

Wheelchair users expressed their perception that taxi drivers did not respect them and showed little concern for them and their needs. This led to some choosing not to use minibus taxis for transport as explained by wheelchair user 1:

‘What makes me sad is when minibus taxi drivers don’t have an idea what I am going through to access my community and they make it difficult to get to places where I need to be. Currently I need to rely on my daughter to push me where I need to be.’ (WCU1 50, male)

Wheelchair user 9 added that taxi drivers did not treat wheelchair users with respect.

‘…most minibus taxi drivers don’t handle us with respect. They don’t have passion for the work and are here just for the money. Yes, some of them just feel nothing for us…Most taxi drivers forgot they are here to deliver a service not only for the so-called normal people but for all of us including wheelchair users. So there needs to be no discrimination from minibus taxi drivers towards wheelchair users.’ (WCU9 37, male)

Disrespectful treatment led to despair. Wheelchair user 6 stated:

‘Some of my experiences with minibus taxi drivers were that a few of them swear and shout at me. I really feel hopeless because of my condition and what makes it worse is when minibus taxi drivers don’t want to assist with transport.’ (WCU6 32, male)

One of the caregivers accused a minibus taxi driver of not providing his services for her parents:

‘I just want to make it clear or get it off my chest that there was a day that you refused to transport my parents. I was so disappointed in the way you treated us and let us down. I realised that day that you felt nothing for us and specifically for my parents who were so in need of transport. It’s these types of negative attitudes which let a person feel vulnerable and not valued. I saw the disappointment and hopelessness on my parents faces and it breaks my heart to see them like that.’ (C8 41, female)

Minibus taxi drivers acknowledged their need to make a living might influence their choice not to provide transport for wheelchair users. In the words of minibus taxi driver 7:

‘As you all know it is about making money and putting bread on the table so the easiest way or the less stress to make money works for me and if I don’t need to pick up wheelchair users so let it be it; sorry for sounding so insensitive but I need to take care of my family.’ (TD7 37, male)

Minibus taxi drivers further argued that the attitudes of wheelchair users were part of the problem. They even claimed some wheelchair users are arrogant and rude:

‘Wheelchair users and their caregivers should also work on their negative attitude. There are some wheelchair users who are also very rude and expect that we need to jump every time for them.’ (TD2 32, male)

He was supported by taxi driver 6 who said:

‘Some wheelchair users are just too entitled, arrogant and rude and they wanted to be treated with respect but don’t show respect to me as a minibus taxi driver.’ (TD 6 38, male)

Wheelchair users 5 and 8 agreed that respect was a two-way street:

‘To get a positive attitude from minibus taxi drivers the wheelchair users should also have a positive attitude. If the wheelchair user is going to be arrogant, I can guarantee you the minibus taxi driver will also be arrogant.’ (WCU5 55, male)

‘It is a give and take if you treat minibus taxi drivers with respect, they might handle us with respect.’ (WCU8 67, male)

It seems that most of the fellow-commuters had negative attitudes towards wheelchair users and were unsure how to interact with wheelchair users. Discomfort and fear might have driven the negative attitudes at least some of the time:

‘As a wheelchair user my experience with fellow-commuters is that they are not very friendly and will not assist me. It’s almost like they don’t know how to communicate with me and are very tense in some cases almost like they feel uncomfortable in my presence.’ (WCU4 57, male)

‘My experiences with follow-commuters were that they think that I am unable to talk or to communicate and act like I don’t exist. I also think some fellow-commuters believe the myths of wheelchair users like if they get to close to wheelchair users that they might become like one of us.’ (WCU1 50, male)

Wheelchair user 3 argued that the negative reactions from fellow commuters might be driven by an emotional reaction to what they perceive as the plight of the wheelchair user:

‘Maybe some of the fellow-commuters they don’t want to engage with wheelchair users because maybe they might feel too emotional and will rather prefer not to interact with the wheelchair users.’ (WCU3 52, male)

Other wheelchair users like wheelchair user 8 experienced the opposite:

‘I had so far the totally opposite experience and the fellow-commuters with whom I have travelled with were very friendly and made me feel welcome in the minibus taxi.’ (WCU8 67, male)

From the thoughts of wheelchair user 6 it seems that the attitude of the wheelchair user can influence that of fellow commuters:

‘My experiences as a wheelchair user were that most people will talk with me because I have a positive attitude so it seems if one has a positive attitude more people like in this case the fellow-commuters will communicate with you as wheelchair user.’ (WCU6 32, male)

#### Actions

Some minibus taxi drivers chose to provide transport to wheelchair users even if they felt unsure or unwilling, even to the point of dropping the person off at home rather than the usual drop off point:

‘…the day when I saw that all minibus taxi drivers unwillingness to transport one of the wheelchair users at a minibus taxi rank. Something inside was just moved and I told myself if I am also just going to ignore this wheelchair users how will he get to his home and also what about his safety because it was getting late. So, I took him to his house. I had a feeling of sadness or guilt but when I dropped him at his house, I saw the happiness and relief on his face. He thanked me and I believed he was happy.’ (TD2 32, male)

Others provided services with a more positive attitude but still describe the challenges related to it:

‘I told you the story about the wheelchair user and his mother I had to transport to their home. I have transported them with love and passion and did not think twice to assist them. It was very difficult for me to transport because his wheelchair was next to me and I struggled to shift the gears because the wheelchair was in the way. But we got safe to their home, and I think I handled the situation very well.’ (TD5 48, male)

For minibus taxi driver 3, a change of heart was facilitated by the health crises of a family member:

‘When one of my family members got a stroke, I realised the challenge we as a family had to transport him for his medical check-up and rehabilitation treatment. This have changed my mind set in so many ways when it comes to the transport of wheelchair users because I knew exactly what they have to go through.’ (TD3 48, male)

Accidents that can be caused by loss of bowel and bladder control negatively impacted the use of minibus taxis. Taxi driver 2 explained:

‘My reason for not providing services for wheelchair users was because of personal hygiene matters. My brother also a minibus taxi driver, once transported a wheelchair user who made a number two [Bowel movement]. I don’t want to deal with these types of aspects because you can just think what it can mean to my business.’ (TD2 32, male)

### Theme 2: Natural, manmade and mechanical environmental barriers

Propelling a wheelchair replaces walking for a wheelchair user. However, propelling the wheelchair in the community was difficult because of natural and manmade environmental barriers. Making use of a wheelchair for community mobility is not the same and not as easy as walking. Importantly, wheelchairs also did not provide a safe or comfortable means to get to minibus taxi pick-up points.

#### Natural environmental barriers

When making use of taxis, the first obstacle participants had to overcome was getting to the pickup points. Propelling the wheelchair on the roads and pavements was tough because wheelchair users did not have the required upper body muscle strength and sustained physical endurance necessary to propel the required distances in often hilly and uneven outdoor environments:

‘I live in an area which is very far from the minibus taxi rank, and I need to cover a great amount of distance to get to the taxi rank.’ (WCU8 67, male)

The hilly nature of the environment also created wheelchair mobility barriers with steep inclines making it difficult for wheelchair users to move around in their communities and to get to the minibus taxi pick up points. Most of them lacked the physical strength to push themselves up and down these steep hills:

‘In the area *where* I am staying is a very huge incline and because of my diagnosis I don’t have the physical strength to push myself up these inclines.’ (WCU6 32, male)

‘I am staying on a hill … I need to go downhill, and the incline is very steep. You can just imagine the fear I am experiencing when going down the hill. Here you need to trust the person who is pushing you otherwise you might land up with some bad consequences.’ (WCU3 52, male)

Not all the roads in this community were tarred, and gravel roads meant mud and pools of water after the rains, in Paarl’s wet winter season:

‘I live in an informal settlement, there are no tarred roads in these informal settlements. I need to push myself on surfaces which are not suitable for wheelchair users at all. I need to go over gravel, stones, and mud.’ (WCU5 55, male)

‘Where I am staying the road is not tarred. After a rainy day I am fearful that my wheels can get stuck in the mud and that I can fall out of the wheelchair.’ (WCU4 57, male)

#### Manmade environmental barriers

Manmade environmental barriers included stairs, pavements, speedbumps and bridges. Participants described not having ramps or lifts at their houses which prevented independent access to streets. Wheelchair user 1 lived on the second floor of a flat with no lift. He was totally dependent on others to carry him down the flights of stairs to get out of the building.

‘I am staying in a flat where there is no lift. I am staying on the 2nd floor and are totally dependent on others to assist me to go down or up the stairs. My barrier starts right here at my home.’ (WCU1 50, male)

Once they leave their homes other barriers prevented or hampered wheelchair mobility and created safety issues. Wheelchair users express their fear when they must push themselves in their wheelchair making use of the road and not the pavements:

‘My issue is the pavements; it is dangerous for us as wheelchair users to make use of the roads because we can be hit at any time by a motor vehicle. The pavements are not accessible because there are no ramps to get on and off the pavements. The municipality could have done these adaptations to the pavement to allow us to have access to the roads.’ (WCU3 52, male)

Others did propel their wheelchair on the road itself but were hindered by the configuration of traffic-calming speed bumps in the road:

‘My challenge is the speed bumps. With my spinal cord injury, I am just not able to push myself over these speed bumps. I tried several times to push myself over them, but the one in my street is just too big. I understand the idea behind the speed bumps so that cars can drive slower, and that people might not be hit by a speeding car, but it makes my life a nightmare.’ (WCU8 67, male)

A specific bridge in the middle of town was also mentioned as a barrier:

‘Then my other concern is the bridge at the main street of Paarl … I have to push myself over that bridge to be in town … and this is my problem or barrier; I need to push myself over the Lady Grey bridge which is very steep. I don’t have the physical strength or the endurance to push myself over that bridge and need assistance from a caregiver of someone else.’ (WCU4 57, male)

#### Mechanical environmental barriers

Because of the physical and manmade barriers presented above, it was difficult for participants to access their community with their wheelchairs. Taxis could help them access the community, but often they could not reach the closest taxi pick-up point without assistance, because of the environmental and manmade challenges described above. Once at the pickup point, they faced mechanical barriers related to the taxi itself. They emphasised the difficulty they experienced during transferring into and out of the taxis. Pointing out the absence of handles at specific, appropriate points inside the minibus taxis:

‘There are just no handrails inside the minibus taxi which makes transfer into and out of the taxi almost impossible.’ (WCU7 54, female)

Not having a handle to hold on during the transfer left the participants with the feeling that they had no control over the activity:

‘…when I am being transferred into the taxi the first thing I am looking or searching for is a handrail to keep my balance and when it is not there it makes me vulnerable getting into or out of the taxi because I don’t feel in control when doing the transfer.’ (WCU5 55, male)

‘It makes it very difficult for me to access the minibus taxi if there are no handles. Imagine you can already not use your legs and now your arms have no handles to hold on which makes you totally dependent on others to assist you.’ (WCU2 49, female)

The minibus taxis had no hydraulic lifts or ramps to assist wheelchair users during boarding:

‘Minibus taxis are not modified to address the need of wheelchair users. Minibus taxis are inaccessible for wheelchair users because it has no ramps or hydraulic lifts to transfer us as wheelchair users into and out of taxis.’ (WCU9 37, male)

Designated taxi ranks also do not have mobile ramps:

‘What I have noticed that at the minibus taxi pick up points there are no special ramps or even temporary ramps which can assist wheelchair users getting into and out of minibus taxis.’ (WCU4 57, male)

Participants felt restricted inside the taxi as they required space to transfer themselves into the taxi or move from one seat to another:

‘When I am inside the minibus taxi, I don’t feel independent or mobile because of the limited space inside the taxi. The space is not big enough to move around inside the minibus taxi. Space is important and need to be considered when dealing with wheelchair users.’ (WCU3 52, male)

Physical impairments like paralysis, contractures, and/or spasticity makes it difficult for wheelchair users to sit inside minibus taxis:

‘Because I have no control over my legs it makes it very difficult and uncomfortable sitting inside a minibus taxi when the space inside is not big enough to accommodate my leg room.’ (WCU6 32, male)

‘The same with me … I am unable to bend my legs, that is why I cannot sit in front seat next to the driver, I need more space for my legs to be accommodated inside the minibus taxi.’ (WCU4 57, male)

### Theme 3: Health and safety concerns

#### Weather conditions

The setting is known to be cold, and wet with poor visibility during winter and very hot during summer. These climatic conditions made participants apprehensive about their health:

‘What is a safety concern for me is the weather conditions in the winter. It is very cold and wet and if you as a wheelchair user need to go to the taxi ranks in the rain it might lead to ‘flu. And especially we with comorbidities it is not a good idea to get sick because of getting wet. I am fearful that a cold can lead to other secondary sicknesses.’ (WCU7 54, female)

‘All that I can add in Paarl it can become very hot. I heard of one of my friends who one day fell out of his wheelchair and there was no one to assist him getting back into his wheelchair. While he was lying on the hot tar waiting for assistance, he picked up 1st to 2nd degree burn wounds. This is the fear I have during summertime that something like this will happen to me.’ (WCU 1 50, male)

Participants also felt that the low visibility in the rain left them vulnerable and at risk.

‘During the wintertime on rainy days when it is dark it makes it very difficult for the traffic to see you if you don’t have reflectors on your wheelchair or reflective clothes.’ (WCU5 55, male)

#### Crime and violence

Another safety concern was related to violence. Crime, violence and gang activities were common in the areas where participants lived:

‘I know how it feels to be mocked, robbed, and assaulted. I woke up inside the hospital. Yes, it is not safe in my neighbourhood.’ (WCU5 55, male)

Gangster activities and shootings worried participants because they were unable to get away quickly when gang fights erupt:

‘I was on my way to the shop when I heard gun shots, everyone was running for their lives except me. I could not get away out of danger quickly. I realised my vulnerability, and hopelessness and that your life is just in the hands of God. I am just thankful I am still alive today, but it was very traumatic for me.’ (WCU4 57, male)

‘The area where I am living there are a lot of gangsters who are tik addicts. I don’t even want to leave my house because they show no mercy and will steal anything for a fix. In the past all my valuables have been stolen and I did not have any insurance on any of those valuables, so it was a total loss for me.’ (WCU7 54, female)

‘Gangsters have no respect for anyone. They don’t discriminate when it comes to crime and will attack or rob you for no reason. This makes me feel very vulnerable; not safe because it will be difficult to defend myself as I am wheelchair bound.’ (WCU4 57, male)

#### Dangerous dogs

Participants further described how they had to deal with dangerous dogs on the streets:

‘My biggest fear is not the gangsters but the dogs. Because the neighbourhood is so dangerous many people keep dogs to protect themselves, their properties, and valuables. This makes it very dangerous to move around in my community with the idea that a dog can come from nowhere and attack you.’ (WCU2 49, female)

The challenges of environmental barriers and safety concerns could have been alleviated by taxis making house calls:

‘Most taxi drivers are not prepared to pick you up or to drop you at your home. I had a very bad experience one day when I was on my way to the pickup point for a taxi when I was robbed and assaulted. This could have been prevented if minibus taxi drivers had considered my safety as a wheelchair user.’ (WCU1 50, male)

#### Reckless driving

**FIGURE 4:**
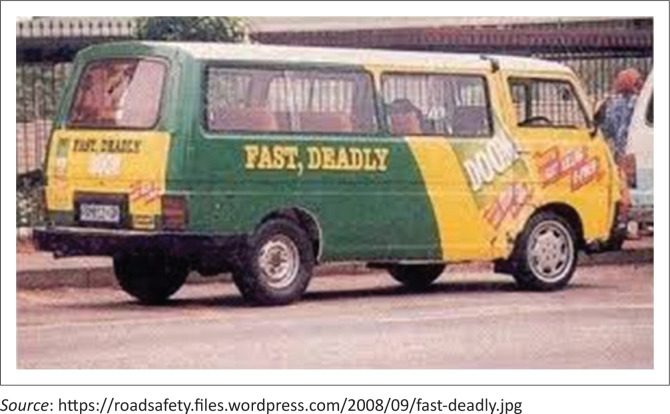
A picture of a taxi with the advertisement referred to by wheelchair user 2.

Using minibus taxis to access the community came with its own safety concerns. Participants had fears related to overloading and speeding which can cause loss of control and an accident:

‘The current minibus taxi services are literally and figuratively like that DOOM advert [*well known household insecticide in South Africa*] “fast and deadly” [*Figure 4*]. We are all aware that most minibus taxi drivers are reckless drivers putting all of our lives in danger … for minibus taxi drivers it is all about making money, so they don’t care when it comes to overloading the minibus taxis in some cases. I must say as a wheelchair user one feels so unsafe because to sit in an overloaded minibus taxi there are so many things that can go wrong if the driver makes an accident. This makes me feel very unsafe.’ (WCU2 49, female)

The lack of handrails to hold on for stability and safety while in motion was also mentioned by some participants:

‘What troubles me the most is when drivers don’t stick to the speed limits. Imagine you don’t have the adequate sitting balance and there are no handles to stabilise you. It is a plan for a disaster to travel in such unsafe conditions.’ (WCU9 37, male)

‘There are no handrails inside the minibus taxi…it makes it very difficult to keep your sitting balance when the taxi is in motion.’ (WCU7)

Erratic driving caused further reservations about safety:

‘Before I was able to take my seat, the driver pulled away so fast that I fell backwards. Luckily for me the seat was just behind me, and I fell unto the seat but what could have happened if the seat was not behind me? I could have hurt myself badly.’ (WCU 6 32, male)

‘Some minibus taxi drivers don’t stop gently they stop at any time or moment to pick up passengers. We as wheelchair users cannot quickly respond to these unexpected ways they are driving.’ (WCU7 54, female)

#### No seat belts

Another major safety issue mentioned was that not all taxis had seatbelts and where seatbelts were available almost no one used them. For wheelchair users who do not have adequate sitting balance, use of seatbelts is a must:

‘Yes, I am also in agreement that with no seat belts and with no handles inside the minibus taxi it is a challenge to maintain your balance especially when the minibus taxis go around corners.’ (WCU1 50, male).

### Theme 4: Poor community participation and quality of life

Poor community mobility because of the interaction between impairments, inaccessible environments, seemingly inappropriate wheelchairs, and inaccessible minibus taxis led to limited participation in community activities. A caregiver indicated that her son, who is a wheelchair user hardly left the house.

‘My son is 45 years old … I would love to take him with me to the shops or sport fields or just get him away from home. The only time when he gets out of the house is when he needs to see a doctor at the hospital for his check-ups…I need to organise other family members or friends to carry him and hire transport which is very expensive.’ (C3 45, female)

Wheelchair user explained that he could not do basic activities that most people take for granted like managing his own money and doing his own shopping:

‘I am totally dependent on my daughter because there are no accessible minibus taxi services, I am no longer in a position to receive my own social grant at the paying points or buying my own groceries at shops.’ (WCU3 52, male)

Wheelchair user 7 mentioned spiritual suffering:

‘What I miss the most is to go to church and to be with my spiritual family and the social interaction I had being part of a spiritual family. The only connection I currently have is when the pastor does home visits, but it is not the same as to worship with your brothers and sisters at the church building.’ (WCU7 54, female)

Whereas, wheelchair user 8 noted his concerns about missing on family life:

‘I would like to see my grandkids when they participate in sport at the schools. Now I only see photos and video clips of them participating in sport which is not the same as being next to the sport fields.’ (WCU8 67, male)

## Discussion

Co-researchers mainly pointed out barriers that made it difficult for wheelchair users to achieve community mobility. These barriers included insufficient knowledge on the part of minibus taxi drivers, negative attitudes, the town’s geography, manmade environmental barriers, gravel roads, inappropriate wheelchairs, winter weather, danger from dogs, criminals and gangsters, as well as reckless driving. In various combinations, these barriers decreased community mobility of wheelchair users and hampered their participation in cherished community activities and thus their quality of life.

Unfortunately, many of the described challenges can be related to lawlessness in the study community. The study community is not an exception in this regard. South Africa, with its famed Constitution, protecting human rights and its numerous laws and policies that are supposed to protect and develop citizens, including those most vulnerable like wheelchair users, fails to uphold and enforce basic laws such as road traffic and dog control rules. More complex phenomena such as gangsterism coupled with crime, murder and drugs have been rife for decades in certain Western Cape communities with government seemingly unable to deal with it. When law enforcement does take a stance violence escalates as was seen in the recent 2023 taxi violence in the Western Cape (Crises 24 2023). The reasons behind this are complex and related to historical inequalities in South African communities caused by apartheid, currently driven by ongoing corruption. Countries like Singapore and Rwanda (Oyamada 2017) have stopped corruption and are prospering. South Africa needs to learn from these examples.

The health and safety concerns raised pose risks to everyone in the community not only wheelchair users. However, wheelchair users are more vulnerable than most because of their impairment and physical limitations. They cannot run away or hide and often lack the physical strength to protect themselves. Cawood and Visagie (2015) showed similar findings, indicating that wheelchair users saw themselves as vulnerable and soft targets for criminals.

Community members have the right to protect themselves and their properties against crime by keeping guard dogs. However, fences can be in disrepair and dogs roam the streets freely; a situation that unfortunately often goes unchecked by local government officials, especially in poorer, more crowded areas of town. It is important that local government enforces regulations regarding dog ownership to ensure that community members are not at risk of being attacked by dogs when out in public.

Similar to, current findings Duri and Luke (2022b) found that drivers of buses and minibus taxis in Tshwane lacked the knowledge to assist wheelchair users and were reluctant to assist them because of fear of hurting them and being sued; perhaps because minibus taxi drivers and fellow commuters have little knowledge of and interaction with wheelchair users. Little understanding of the impairments of wheelchair users and how it affects functioning might have caused unrealistic expectations of what the wheelchair users can and cannot do to get into and out of taxis. Knowledge on how to assist persons with various impairments with transfers and how to fold and store wheelchairs are not intuitive and must be taught (Duri & Luke 2022b).

Not knowing how to provide assistance coupled with the uncomfortable emotions disability often evoke, as eloquently described by Hughes (2020), could have led to the decision of some taxi drivers not to provide transport to wheelchair users. One taxi driver in the current study who did provide transport to a wheelchair user, could not quite label his emotional discomfort, but described it as ‘sadness’ or ‘guilt’. Hughes (2020) explains that emotions, like pity, guilt, fear or even disgust, and not knowing how to act or what to say are common occurrences among persons without disabilities, such as drivers and fellow commuters in the current study, when they encounter persons with disabilities. These uncomfortable emotions might result in people distancing themselves from the person with the disability (Dohmen 2016). Seeing a person with a disability might remind others of their own vulnerability and cause inner tension they would rather avoid (Dohmen 2016). Usually, these reactions can be remedied by education and awareness raising (Dohmen 2016). If not remedied, these emotions can manifest through disrespect and rudeness towards wheelchair users as described in the current findings and shown by Bezyak et al. (2017) and Duri and Luke (2022b). Unfairly, the burden often falls on the person with the disability to reach out and put others at ease.

Identified environmental barriers were mostly manmade. Norms applied in the planning and construction of roads, pavements, bridges and vehicles did not include consideration of universal design principles (Duman & Asilsoy 2022). Poor visibility on cloudy days and at night can have serious consequences for wheelchair users if they do not wear reflectors and do not have access to the pavements. Reckless driving that put pedestrians at risk was not reported by current participants, but is common in South African peri-urban areas. The disregard of universal design was probably not conscious but based on deep-seated ableist thought patterns in our societies (Hamraie 2017). This excludes not only wheelchair users, but also others that use wheeled devices such as parents with strollers, persons with mobility impairments and people using shopping or other carts to transport goods (often used for activities related to livelihood creation) (Duman & Asilsoy 2022). Continued disregard for universal design principles in urban planning and design thus created conditions for social and occupational injustice (Duman & Asilsoy 2022).

Seat belts are a requirement according to law, but most co-researchers indicated that seat belts are seldom or never used in minibus taxis. Not wearing a seatbelt and erratic driving such as fast lane changing and cornering, quick stops and starts make wheelchair users more vulnerable to injury than their abled bodied peers because they are less unable to maintain their sitting balance because of paralysis or weakness of limbs. These experiences draw attention to risks experienced by all minibus taxi passengers. The toll that taxi accidents take in terms of lives lost and morbidity is widely reported in South African media, yet is seemingly not addressed by road traffic officials. The reasons are unclear and require further study. Maleka et al. (2012) indicated that wheelchair users would rather stay at home because of fear in conjunction with the transport problems. It is important for stakeholders to advocate for community safety of wheelchair users and other vulnerable groups such as children and elderly people and explore ways in which the police and neighbourhood watch systems can assist to make the communities safer for all, including wheelchair users.

Travelling in a wheelchair on wet, muddy gravel roads poses additional challenges to the general health risks of cold wet winters. Wheelchair users and those pushing them face slippery conditions, dirty hands and clothes as well as the risk of the wheelchair getting stuck, or tipping. This increased the risk of injuries, because of falling out of the wheelchair, or getting ill, on account of being exposed to wet and cold weather. Bezyak et al. (2017) indicated similar concerns in a North American study. Rainy weather also decreases visibility. But wheelchair users can increase their visibility to motorists with reflectors on the wheelchair, reflective clothing, as well as adding a small flagpole.

None of the wheelchair users were issued with active use wheelchairs with an adjustable centre of gravity, or by centring weight over the rear wheel which enhances mobility and prevents castors from getting stuck. All-terrain models would have been the most appropriate wheelchair considering the physical environment. Wheelchairs with a short wheelbase are not appropriate for negotiating dirt roads, and obstacles like kerbs and speed bumps (Visagie et al. 2015). Motorised wheelchairs should be considered (bearing in mind increased costs) for wheelchair users who lack the endurance and/or physical power and muscle control to master basic mobility skills to independently access the community (Visagie et al. 2023).

## Strengths and limitations

Co-operative group members were passionate about the topic and aligned themselves with the objectives of the study and shared ownership of the process. Data were collected directly after South Africa came out of a prolonged coronavirus disease 2019 (COVID-19) lockdown. Some wheelchair users, mostly those who were older, who were approached to participate in the study were afraid of contracting the disease and therefore chose not to participate. This decreased the age range of participants. However, those who did participate had a variety of diagnoses and thus differing abilities, which meant a spectrum of experiences could be explored.

## Conclusion, recommendations and next steps

### Recommendations

Recommendations are made keeping national and international policy such as to the United Nations Convention on the Rights of Persons with Disabilities (UNCRPD) in mind.

- As also recommended by Duri and Lake (2022b), workshops with minibus taxi drivers and wheelchair users together should be done to work on respect, understanding and communicating with each other and to explore optimal ways for wheelchair users to board and disembark as well as safety during transit. Addressing stigma around disability through open dialogue both with taxi drivers and other community members may also be beneficial.
- It is important that wheelchair users together with other vulnerable groups and health workers emphasise to city planners that all roads especially those in informal settlements, where people move about on foot or in wheelchairs and/or bicycles, should be tarred, and pavements should be smooth with kerb cuts. Accessible safe pedestrian crossings should be available.
- This study again highlights the need for universal design in transport infrastructure and that the roll-out therefore, which currently is limited to a few major South African cities, should be accelerated.
- Placing sturdy rails or grab bars at strategic points throughout the taxi is something manufacturers can be made aware of. This will benefit wheelchair users as well as all commuters. Minibus taxi drivers, wheelchair users and their caregivers should ensure that seatbelts are always secured when making use of minibus taxis.
- Lobby for availability of a range of wheelchairs to enable wheelchair users to be fitted with the most appropriate wheelchair meeting their functional and environmental needs. This may include provision of more than one wheelchair to allow community access and upskilling therapists and education of procurement and finance teams.
- To enhance safety, it is recommended that wheelchair users always ensure someone knows their travel plans, have a cell phone that can be tracked and which is programmed with emergency numbers. If possible, they should not travel alone.
- Existing legislation regarding road traffic laws, dog ownership, violence and crime must be enforced.

### Conclusion

This study provides a variation on a story often told, but no less important because of its familiarity. The challenges facing wheelchair users when accessing minibus taxis have been well described in literature; however, the views of minibus taxi drivers are not usually included. Their reality can be further explored in future studies. Additionally, the link between poor community mobility and community participation has not often been made in African research. Finally, the unfortunate conclusion that several of the obstacles, danger, and emotional upheaval are caused by a general state of lawlessness in South African communities is an unpalatable truth that has become such an integral part of South African life that it is hardly noticed.

### Next steps

This article reports on the first step of the inquiry. In further phases, different strategies and cycles were planned, actively tried, and then reflected on to deal with some of the identified challenges. The findings included: transfer techniques, ways of storing the wheelchair in the minibus taxi, knowledge building and awareness raising, home pick-up and drop-off, as well as subsidising different access features such as ramps or a hoist and docking station in a few minibus taxis. These will be described in future articles.
